# *trans*-Bis[acetone (2-hydroxy­benzo­yl)hydrazonato-κ^2^ *N*′,*O*]bis­(pyridine-κ*N*)zinc(II)

**DOI:** 10.1107/S1600536809001949

**Published:** 2009-01-23

**Authors:** Ming-Xing Yang, Shen Lin, Li-Juan Chen, Xiao-Hua Chen

**Affiliations:** a College of Chemistry and Materials Science, Fujian Normal University, Fuzhou, Fujian 350007, People’s Republic of China; b State Key Laboratory of Structural Chemistry, Fujian Institute of Research on the Structure of Matter, Chinese Academy of Sciences, Fuzhou, Fujian 350002, People’s Republic of China

## Abstract

In the title compound, [Zn(C_10_H_11_N_2_O_2_)_2_(C_5_H_5_N)_2_], the Zn^II^ atom lies on an inversion centre, and is coordinated in a distorted octa­hedral geometry by two carbonyl O atoms and two imino N atoms from two anionic bidentate acetone (2-hydroxy­benzo­yl)hydrazone ligands and by two N atoms from two pyridine mol­ecules. The hydroxyl group acts as a donor, forming an intra­molecular O—H⋯N hydrogen bond.

## Related literature

For general background, see: Bai *et al.* (2006 ▶); Gao *et al.* (1998 ▶); Grove *et al.* (2004 ▶); Liu & Gao (1998 ▶); Ma *et al.* (1989 ▶). For related structures, see: Chen & Liu (2004 ▶); Domiano *et al.* (1975 ▶); Hu *et al.* (2006 ▶, 2007 ▶); Li *et al.* (2006 ▶); Liu *et al.* (1999 ▶); Samanta *et al.* (2007 ▶); Wen *et al.* (2000 ▶); Wu *et al.* (2006 ▶); Xiao *et al.* (2000 ▶).
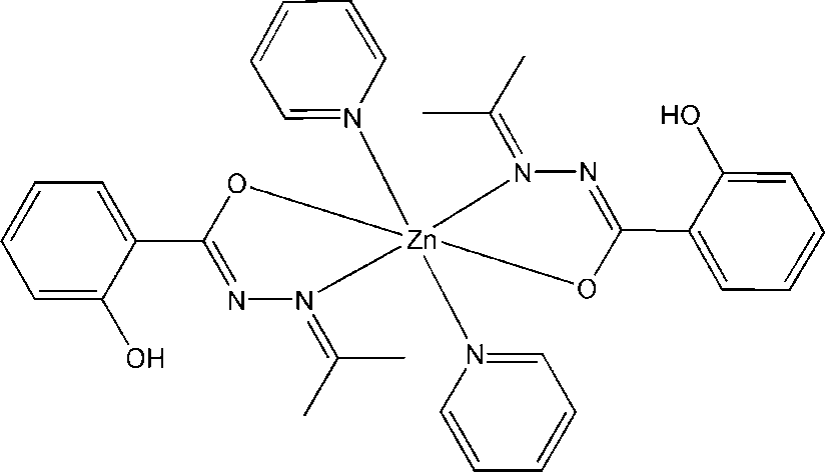


## Experimental

### 

#### Crystal data

[Zn(C_10_H_11_N_2_O_2_)_2_(C_5_H_5_N)_2_]
*M* *_r_* = 605.99
Monoclinic, 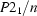

*a* = 7.8225 (8) Å
*b* = 10.0381 (10) Å
*c* = 18.8201 (18) Å
β = 96.21 (4)°
*V* = 1469.1 (3) Å^3^
*Z* = 2
Mo *K*α radiation
μ = 0.88 mm^−1^
*T* = 293 (2) K
0.35 × 0.26 × 0.15 mm

#### Data collection

Rigaku R-AXIS RAPID diffractometer
Absorption correction: none
13028 measured reflections
3282 independent reflections
2334 reflections with *I* > 2σ(*I*)
*R*_int_ = 0.034

#### Refinement

*R*[*F* ^2^ > 2σ(*F* ^2^)] = 0.033
*wR*(*F* ^2^) = 0.097
*S* = 0.98
3282 reflections
188 parameters
H-atom parameters constrained
Δρ_max_ = 0.38 e Å^−3^
Δρ_min_ = −0.33 e Å^−3^

### 

Data collection: *PROCESS-AUTO* (Rigaku, 1998 ▶); cell refinement: *PROCESS-AUTO*; data reduction: *PROCESS-AUTO*; program(s) used to solve structure: *SHELXS97* (Sheldrick, 2008 ▶); program(s) used to refine structure: *SHELXL97* (Sheldrick, 2008 ▶); molecular graphics: *SHELXTL* (Sheldrick, 2008 ▶); software used to prepare material for publication: *SHELXTL*.

## Supplementary Material

Crystal structure: contains datablocks global, I. DOI: 10.1107/S1600536809001949/hy2173sup1.cif

Structure factors: contains datablocks I. DOI: 10.1107/S1600536809001949/hy2173Isup2.hkl

Additional supplementary materials:  crystallographic information; 3D view; checkCIF report

## Figures and Tables

**Table 1:** Selected bond lengths (Å)

Zn1—O2	2.0319 (14)
Zn1—N2	2.1912 (16)
Zn1—N3	2.3013 (18)

**Table 2:** Hydrogen-bond geometry (Å, °)

*D*—H⋯*A*	*D*—H	H⋯*A*	*D*⋯*A*	*D*—H⋯*A*
O1—H1⋯N1	0.99	1.61	2.535 (2)	154

**Fig. 1.**
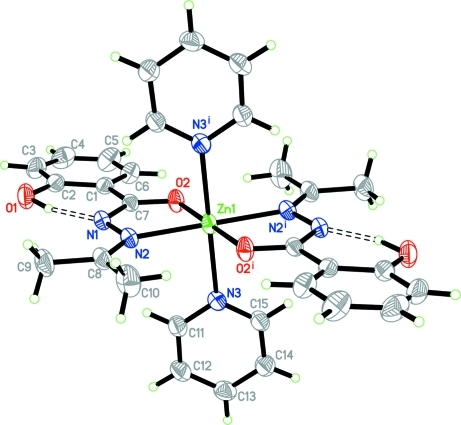
Molecular structure of the title compound. Displacement ellipsoids are drawn at the 30% probability level. Hydrogen bonds are shown as dashed lines. [Symmetry code: (i) 1 - x, -y, -z.]

## supplementary crystallographic information

### Comment

Hydrazones have been attracting much attention by chemists in recent years because of their biological activities, chemical and industrial versatility, and strong tendency to chelate transition metals (Bai *et al.*, 2006; Grove *et al.*, 2004), lanthanide metals (Ma *et al.*, 1989) and main group metals (Gao *et al.*, 1998; Liu & Gao, 1998). In particular, salicyloylhydrazone can be very flexible and finely tuned at the molecular level to take versatile bonding modes. It can act as a bi-, tri-, tetra- and even pentadentate ligand. A number of zinc(II) complexes with salicyloylhydrazone ligands have been studied (Hu *et al.*, 2006; Hu *et al.*, 2007; Li *et al.*, 2006; Samanta *et al.*, 2007; Wu *et al.*, 2006). As an extension of the work on the structural characterization of salicyloylhydrazone complexes, the preparation and crystal structure of the title zinc(II) complex are reported here.

The molecular structure of the title compound is shown in Fig. 1. The Zn^II^ atom lies on an inversion centre and has an axially elongated octahedral coordination geometry. The two carbonyl O atoms and the two imino N atoms make up the equatorial plane and the two N atoms of two pyridine molecules occupy the axial positions at longer distances (Table 1). Double-bond character is present in C7—N1 and C8—N2, as judged from their bond lengths [1.322 (2) and 1.286 (2) Å] (Domiano *et al.*, 1975; Liu *et al.*, 1999; Xiao *et al.*, 2000). The C7—O2 bond length of 1.273 (2) Å approaches the value of 1.263 Å expected for an enolic form of the hydrazone ligand (Chen & Liu, 2004; Wen *et al.*, 2000). The data suggest enolization and deprotonation of the hydrazone groups, which is different from the analogous Zn^II^ complex with the same ligand (Li *et al.*, 2006). There exists an intramolecular O—H···N hydrogen bond (Table 2).

### Experimental

All reagents were commercially available and of analytical grade. To a solution of Zn(CH_3_COO)_2_.2H_2_O (0.110 g, 0.5 mmol) in pyridine (5 ml) was slowly added a suspension of acetone-N-salicyloylhydrazone (0.192 g, 1.0 mmol) in DMF(5 ml). The resulting red solution was stirred for 20 min and then filtered. After standing for 5 d, yellow crystals were separated from the filtrate.

### Refinement

H atoms bonded to C atoms were positioned geometrically and refined as riding atoms, with C—H = 0.93 (aromatic) and 0.96 (CH_3_) Å and with *U*_iso_(H) = *xU*_eq_(C), where *x*=1.2 for aromatic and 1.5 for methyl H atoms. H atom of the hydroxyl group was located in difference Fourier map and refined isotropically with its coordinates fixed.

### Crystal data

**Table:**

[Zn(C_10_H_11_N_2_O_2_)_2_(C_5_H_5_N)_2_]	*F*(000) = 632
*M**_r_* = 605.99	*D*_x_ = 1.370 Mg m^−^^3^
Monoclinic, *P*2_1_/*n*	Mo *K*α radiation, λ = 0.71073 Å
Hall symbol: -P 2yn	Cell parameters from 3282 reflections
*a* = 7.8225 (8) Å	θ = 2.3–27.5°
*b* = 10.0381 (10) Å	µ = 0.88 mm^−^^1^
*c* = 18.8201 (18) Å	*T* = 293 K
β = 96.21 (4)°	Block, yellow
*V* = 1469.1 (3) Å^3^	0.35 × 0.26 × 0.15 mm
*Z* = 2	

### Data collection

**Table:**

Rigaku R-AXIS RAPID diffractometer	2334 reflections with *I* > 2σ(*I*)
Radiation source: 18 kW rotation anode	*R*_int_ = 0.034
graphite	θ_max_ = 27.5°, θ_min_ = 2.3°
ω scans	*h* = 0→10
13028 measured reflections	*k* = 0→13
3282 independent reflections	*l* = −24→24

### Refinement

**Table:**

Refinement on *F*^2^	Primary atom site location: structure-invariant direct methods
Least-squares matrix: full	Secondary atom site location: difference Fourier map
*R*[*F*^2^ > 2σ(*F*^2^)] = 0.033	Hydrogen site location: inferred from neighbouring sites
*wR*(*F*^2^) = 0.097	H-atom parameters constrained
*S* = 0.98	*w* = 1/[σ^2^(*F*_o_^2^) + (0.058*P*)^2^] where *P* = (*F*_o_^2^ + 2*F*_c_^2^)/3
3282 reflections	(Δ/σ)_max_ < 0.001
188 parameters	Δρ_max_ = 0.38 e Å^−^^3^
0 restraints	Δρ_min_ = −0.33 e Å^−^^3^

### Special details

**Table:**

Geometry. All e.s.d.'s (except the e.s.d. in the dihedral angle between two l.s. planes) are estimated using the full covariance matrix. The cell e.s.d.'s are taken into account individually in the estimation of e.s.d.'s in distances, angles and torsion angles; correlations between e.s.d.'s in cell parameters are only used when they are defined by crystal symmetry. An approximate (isotropic) treatment of cell e.s.d.'s is used for estimating e.s.d.'s involving l.s. planes.

### Fractional atomic coordinates and isotropic or equivalent isotropic displacement parameters (Å^2^)

**Table:**

	*x*	*y*	*z*	*U*_iso_*/*U*_eq_	
Zn1	0.5000	0.0000	0.0000	0.04214 (12)	
O1	0.4610 (2)	0.49143 (15)	0.11896 (11)	0.0690 (5)	
H1	0.5171	0.4215	0.0926	0.113 (11)*	
O2	0.35166 (17)	0.09309 (14)	0.06705 (8)	0.0492 (3)	
N1	0.5358 (2)	0.27234 (16)	0.06295 (8)	0.0423 (4)	
N2	0.6294 (2)	0.19207 (16)	0.02003 (8)	0.0433 (4)	
N3	0.6638 (2)	−0.09222 (17)	0.09654 (9)	0.0469 (4)	
C1	0.2948 (2)	0.2914 (2)	0.12902 (10)	0.0435 (4)	
C2	0.3302 (3)	0.4273 (2)	0.14421 (11)	0.0525 (5)	
C3	0.2284 (4)	0.4958 (3)	0.18860 (14)	0.0694 (7)	
H3A	0.2484	0.5859	0.1977	0.083*	
C4	0.1004 (4)	0.4326 (3)	0.21861 (15)	0.0755 (8)	
H4A	0.0372	0.4790	0.2496	0.091*	
C5	0.0628 (4)	0.3007 (3)	0.20383 (15)	0.0753 (7)	
H5A	−0.0267	0.2587	0.2237	0.090*	
C6	0.1600 (3)	0.2321 (2)	0.15905 (12)	0.0567 (5)	
H6A	0.1342	0.1433	0.1487	0.068*	
C7	0.3987 (2)	0.21174 (19)	0.08292 (10)	0.0397 (4)	
C8	0.7671 (3)	0.2453 (2)	0.00115 (12)	0.0511 (5)	
C9	0.8255 (3)	0.3833 (2)	0.02229 (16)	0.0741 (7)	
H9A	0.7457	0.4226	0.0515	0.111*	
H9B	0.8308	0.4364	−0.0198	0.111*	
H9C	0.9374	0.3792	0.0487	0.111*	
C10	0.8761 (3)	0.1689 (3)	−0.04426 (17)	0.0835 (9)	
H10A	0.8262	0.0826	−0.0543	0.125*	
H10B	0.9893	0.1587	−0.0195	0.125*	
H10C	0.8833	0.2159	−0.0883	0.125*	
C11	0.7532 (3)	−0.0149 (2)	0.14528 (13)	0.0584 (6)	
H11A	0.7448	0.0771	0.1401	0.070*	
C12	0.8567 (3)	−0.0653 (3)	0.20266 (13)	0.0659 (7)	
H12A	0.9172	−0.0082	0.2352	0.079*	
C13	0.8699 (3)	−0.2014 (3)	0.21143 (13)	0.0647 (6)	
H13A	0.9398	−0.2380	0.2496	0.078*	
C14	0.7773 (3)	−0.2813 (2)	0.16238 (13)	0.0624 (6)	
H14A	0.7824	−0.3735	0.1667	0.075*	
C15	0.6764 (3)	−0.2228 (2)	0.10655 (12)	0.0549 (5)	
H15A	0.6134	−0.2781	0.0739	0.066*	

### Atomic displacement parameters (Å^2^)

**Table:**

	*U*^11^	*U*^22^	*U*^33^	*U*^12^	*U*^13^	*U*^23^
Zn1	0.04131 (17)	0.03917 (18)	0.04726 (19)	−0.01003 (14)	0.01086 (12)	−0.00788 (15)
O1	0.0754 (11)	0.0435 (8)	0.0881 (12)	−0.0109 (8)	0.0086 (10)	−0.0158 (9)
O2	0.0496 (7)	0.0406 (7)	0.0606 (9)	−0.0129 (6)	0.0201 (6)	−0.0087 (7)
N1	0.0429 (8)	0.0400 (8)	0.0437 (8)	−0.0079 (7)	0.0031 (7)	−0.0041 (7)
N2	0.0410 (8)	0.0441 (9)	0.0447 (9)	−0.0106 (7)	0.0046 (7)	−0.0030 (7)
N3	0.0466 (9)	0.0486 (10)	0.0449 (9)	−0.0068 (8)	0.0024 (7)	−0.0018 (8)
C1	0.0495 (10)	0.0437 (10)	0.0360 (9)	0.0032 (9)	−0.0008 (8)	−0.0007 (8)
C2	0.0589 (12)	0.0486 (12)	0.0476 (12)	0.0048 (11)	−0.0056 (10)	−0.0054 (10)
C3	0.0841 (17)	0.0553 (14)	0.0669 (15)	0.0167 (14)	−0.0013 (13)	−0.0165 (13)
C4	0.0821 (18)	0.0784 (19)	0.0683 (16)	0.0286 (16)	0.0177 (14)	−0.0098 (14)
C5	0.0811 (17)	0.0750 (18)	0.0756 (17)	0.0154 (15)	0.0344 (14)	0.0028 (14)
C6	0.0617 (13)	0.0532 (13)	0.0582 (13)	0.0043 (11)	0.0201 (10)	0.0016 (11)
C7	0.0435 (9)	0.0391 (9)	0.0355 (9)	−0.0050 (8)	−0.0003 (7)	−0.0001 (8)
C8	0.0447 (10)	0.0505 (12)	0.0587 (12)	−0.0168 (9)	0.0076 (9)	0.0020 (10)
C9	0.0645 (14)	0.0572 (15)	0.102 (2)	−0.0289 (12)	0.0137 (14)	0.0001 (14)
C10	0.0660 (15)	0.0813 (19)	0.111 (2)	−0.0259 (14)	0.0433 (15)	−0.0134 (17)
C11	0.0641 (13)	0.0516 (13)	0.0571 (13)	−0.0078 (11)	−0.0041 (11)	−0.0051 (10)
C12	0.0678 (15)	0.0684 (16)	0.0573 (14)	−0.0087 (13)	−0.0130 (12)	−0.0074 (12)
C13	0.0652 (14)	0.0717 (16)	0.0536 (13)	−0.0039 (13)	−0.0107 (11)	0.0098 (12)
C14	0.0699 (15)	0.0526 (13)	0.0626 (14)	−0.0059 (12)	−0.0032 (12)	0.0078 (12)
C15	0.0588 (12)	0.0526 (12)	0.0511 (12)	−0.0104 (11)	−0.0039 (10)	−0.0007 (10)

### Geometric parameters (Å, °)

**Table:**

Zn1—O2^i^	2.0319 (14)	C4—H4A	0.9300
Zn1—O2	2.0319 (14)	C5—C6	1.379 (3)
Zn1—N2	2.1912 (16)	C5—H5A	0.9300
Zn1—N2^i^	2.1912 (16)	C6—H6A	0.9300
Zn1—N3^i^	2.3013 (18)	C8—C10	1.485 (3)
Zn1—N3	2.3013 (18)	C8—C9	1.498 (3)
O1—C2	1.338 (3)	C9—H9A	0.9600
O1—H1	0.99	C9—H9B	0.9600
O2—C7	1.273 (2)	C9—H9C	0.9600
N1—C7	1.322 (2)	C10—H10A	0.9600
N1—N2	1.402 (2)	C10—H10B	0.9600
N2—C8	1.286 (2)	C10—H10C	0.9600
N3—C15	1.326 (3)	C11—C12	1.374 (3)
N3—C11	1.339 (3)	C11—H11A	0.9300
C1—C6	1.384 (3)	C12—C13	1.379 (4)
C1—C2	1.415 (3)	C12—H12A	0.9300
C1—C7	1.485 (3)	C13—C14	1.369 (3)
C2—C3	1.396 (3)	C13—H13A	0.9300
C3—C4	1.359 (4)	C14—C15	1.375 (3)
C3—H3A	0.9300	C14—H14A	0.9300
C4—C5	1.378 (4)	C15—H15A	0.9300
			
O2^i^—Zn1—O2	180.00 (6)	C6—C5—H5A	120.5
O2^i^—Zn1—N2	103.14 (6)	C5—C6—C1	122.0 (2)
O2—Zn1—N2	76.86 (6)	C5—C6—H6A	119.0
O2^i^—Zn1—N2^i^	76.86 (6)	C1—C6—H6A	119.0
O2—Zn1—N2^i^	103.14 (6)	O2—C7—N1	125.93 (18)
N2—Zn1—N2^i^	180.00 (9)	O2—C7—C1	118.57 (17)
O2^i^—Zn1—N3^i^	90.07 (6)	N1—C7—C1	115.49 (17)
O2—Zn1—N3^i^	89.93 (6)	N2—C8—C10	119.64 (19)
N2—Zn1—N3^i^	89.38 (6)	N2—C8—C9	123.4 (2)
N2^i^—Zn1—N3^i^	90.62 (6)	C10—C8—C9	116.92 (19)
O2^i^—Zn1—N3	89.93 (6)	C8—C9—H9A	109.5
O2—Zn1—N3	90.07 (6)	C8—C9—H9B	109.5
N2—Zn1—N3	90.62 (6)	H9A—C9—H9B	109.5
N2^i^—Zn1—N3	89.38 (6)	C8—C9—H9C	109.5
N3^i^—Zn1—N3	180.00 (10)	H9A—C9—H9C	109.5
C2—O1—H1	103.6	H9B—C9—H9C	109.5
C7—O2—Zn1	113.95 (12)	C8—C10—H10A	109.5
C7—N1—N2	112.91 (15)	C8—C10—H10B	109.5
C8—N2—N1	115.16 (17)	H10A—C10—H10B	109.5
C8—N2—Zn1	134.68 (15)	C8—C10—H10C	109.5
N1—N2—Zn1	110.16 (11)	H10A—C10—H10C	109.5
C15—N3—C11	116.73 (19)	H10B—C10—H10C	109.5
C15—N3—Zn1	122.43 (14)	N3—C11—C12	123.0 (2)
C11—N3—Zn1	120.83 (15)	N3—C11—H11A	118.5
C6—C1—C2	118.3 (2)	C12—C11—H11A	118.5
C6—C1—C7	119.76 (18)	C11—C12—C13	119.3 (2)
C2—C1—C7	121.98 (19)	C11—C12—H12A	120.4
O1—C2—C3	118.9 (2)	C13—C12—H12A	120.4
O1—C2—C1	122.2 (2)	C14—C13—C12	118.2 (2)
C3—C2—C1	119.0 (2)	C14—C13—H13A	120.9
C4—C3—C2	120.8 (2)	C12—C13—H13A	120.9
C4—C3—H3A	119.6	C13—C14—C15	118.9 (2)
C2—C3—H3A	119.6	C13—C14—H14A	120.6
C3—C4—C5	121.0 (3)	C15—C14—H14A	120.6
C3—C4—H4A	119.5	N3—C15—C14	123.9 (2)
C5—C4—H4A	119.5	N3—C15—H15A	118.0
C4—C5—C6	118.9 (3)	C14—C15—H15A	118.0
C4—C5—H5A	120.5		

Symmetry codes: (i) −*x*+1, −*y*, −*z*.

### Hydrogen-bond geometry (Å, °)

**Table:**

*D*—H···*A*	*D*—H	H···*A*	*D*···*A*	*D*—H···*A*
O1—H1···N1	0.99	1.61	2.535 (2)	154
